# Unclear relationships between mean survival rate and its environmental variance in vertebrates

**DOI:** 10.1002/ece3.11104

**Published:** 2024-03-03

**Authors:** Tomas Pärt, Tobias Jeppsson, Matthieu Paquet, Debora Arlt, Ane T. Laugen, Matthew Low, Jonas Knape, Anna Qvarnström, Pär Forslund

**Affiliations:** ^1^ Department of Ecology Swedish University of Agricultural Sciences Uppsala Sweden; ^2^ KTH, Royal Institute of Technology Stockholm Sweden; ^3^ Institute of Mathematics of Bordeaux, CNRS University of Bordeaux Talence France; ^4^ Theoretical and Experimental Ecology Station (SETE) CNRS Moulis France; ^5^ SLU Swedish Species Information Centre Swedish University of Agricultural Sciences Uppsala Sweden; ^6^ Department of Natural Sciences University of Agder Kristiansand Norway; ^7^ Department of Animal Ecology EBC Uppsala Sweden

**Keywords:** annual survival rates, demographic buffering, demographic lability, environmental canalization, environmental fluctuations, environmental stochasticity, process variance, stochastic population growth, temporal variation

## Abstract

Current environmental changes may increase temporal variability of life history traits of species thus affecting their long‐term population growth rate and extinction risk. If there is a general relationship between environmental variances (EVs) and mean annual survival rates of species, that relationship could be used as a guideline for analyses of population growth and extinction risk for populations, where data on EVs are missing. For this purpose, we present a comprehensive compilation of 252 EV estimates from 89 species belonging to five vertebrate taxa (birds, mammals, reptiles, amphibians and fish) covering mean annual survival rates from 0.01 to 0.98. Since variances of survival rates are constrained by their means, particularly for low and high mean survival rates, we assessed whether any observed relationship persisted after applying two types of commonly used variance stabilizing transformations: relativized EVs (observed/mathematical maximum) and logit‐scaled EVs. With raw EVs at the arithmetic scale, mean–variance relationships of annual survival rates were hump‐shaped with small EVs at low and high mean survival rates and higher (and widely variable) EVs at intermediate mean survival rates. When mean annual survival rates were related to relativized EVs the hump‐shaped pattern was less distinct than for raw EVs. When transforming EVs to logit scale the relationship between mean annual survival rates and EVs largely disappeared. The within‐species juvenile‐adult slopes were mainly positive at low (<0.5) and negative at high (>0.5) mean survival rates for raw and relativized variances while these patterns disappeared when EVs were logit transformed. Uncertainties in how to interpret the results of relativized and logit‐scaled EVs, and the observed high variation in EV's for similar mean annual survival rates illustrates that extrapolations of observed EVs and tests of life history drivers of survival–EV relationships need to also acknowledge the large variation in these parameters.

## Graphical abstract

The relationship between mean survival rate and its temporal environmental (process) variance is dependent on the mathematical scaling.

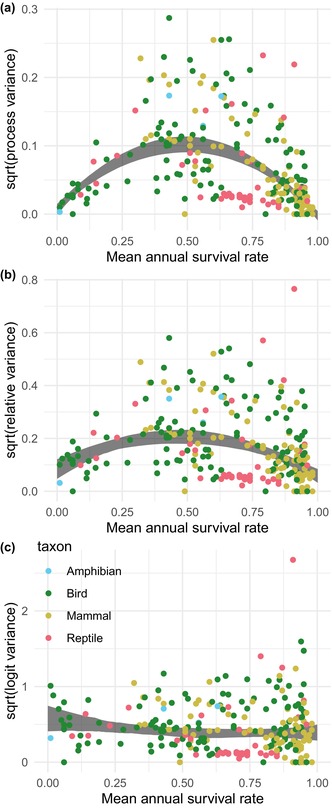

## 1 INTRODUCTION

Recent and ongoing anthropogenic environmental changes, such as global warming, are resulting in increased environmental fluctuations (IPCC, 2022). Such fluctuations in the external environment causes temporal variation in demographic parameters (e.g. annual survival rate and reproduction) over time, from here on referred to as environmental variance (EV, also denoted as ‘process variance’). The environmental variance, as well as the temporal mean, of demographic rates (together with environmental covariance among these parameters; see Le Coeur et al., 2022) are instrumental for understanding and predicting long‐term dynamics of populations, including estimating extinction risks of species and populations (Boyce et al., 2006). For many species under threat estimates of demographic rates are missing because the long and detailed time series required for estimating them are rare. A compilation of EVs covering a wide range of life history types could reveal general relationships of EVs in vital rates in relation to life history types, which could give realistic guidelines to model scenarios of long‐term stochastic population growth rates and extinction risks when such data are lacking.

Previous compilations of demographic rates and their EVs have often considered survival (see, e.g. Hilde et al., 2020) partly because survival is measured along the same simple binary yardstick while other demographic parameters (e.g. reproduction) are measured in many different ways and on different scales. Another reason is that most published estimates of EVs in demographic rates concerns survival rates. The question is whether we should expect a relationship between EVs and mean survival rates and whether EVs are similar among species sharing similar mean survival rates.

There are at least two major reasons to why EVs may vary with mean survival of a species. The first reason is that variances are bound to be related to the mean for survival rates as the variance is strongly constrained when a mean survival rate is close to either 0 or 1. Thus, without any other constraints, the mathematical expectation is to observe the highest EVs for survival rates close to 0.5 and the lowest close to 0 or 1. The second reason is that EVs could relate to mean survival as a result of past life history evolution. Increased EVs generally lead to reduced stochastic growth rates if mean demographic rates are held constant (see, e.g. Koons et al., 2009; Tuljapurkar, 1982). It has therefore been hypothesized that natural selection could favour buffering against EVs (the ‘demographic buffering hypothesis’; Gillespie, 1977; Pfister, 1998). However, depending on the functional relationship between environmental conditions and demographic parameters, increased variation in environmental conditions can under some conditions (Barraquand & Yoccoz, 2013) lead to increased stochastic growth rates (via an increase in mean demographic rates). This has led to the alternative possibility that selection could sometimes favour selection for demographic parameters that exhibit plasticity to positively track variations in the environment, the ‘demographic lability’ hypothesis. Contrary to the demographic buffering hypothesis, the demographic lability hypothesis predicts a positive correlation between importance and variability of demographic traits (Koons et al., 2009).

Explicit testing of the buffering and lability hypotheses requires data on sensitivities/elasticities and EVs of demographic parameters, such as mean survival rates. Empirical studies suggest that variation in mean adult survival rates at least to some extent may positively relate to sensitivities/elasticities across species (see, e.g. Gaillard & Yoccoz, 2003; Sæther & Bakke, 2000). Based on simulated data, Koons et al. (2009) suggested that natural selection may be more likely to favour lability in survival (i.e. high EVs) in species with low (<0.5) mean survival rates and buffering in species with high (>0.5) mean survival rates. Similarly, Le Coeur et al. (2022) found demographic lability in demographic parameters to be more likely among fast‐life history species while demographic buffering was more likely among slow‐living species. Thus, even when lacking sensitivities/elasticities of survival, we may, for example, expect species with high mean survival rates to display reduced EVs compared to those characterized by intermediate survival rates. The question is then how to investigate whether EVs vary in relation to mean survival rates while accounting for the above‐mentioned mathematical constraint of variance–mean relationships. To avoid the constraint of variance–mean relationship of survival two transformations have been suggested, namely ‘relativized variance’ (i.e. the scaling of observed variance to the mathematical maximum; Gaillard & Yoccoz, 2003; Morris & Doak, 2004) and logit‐transformed variance (i.e. the variance of logit‐transformed survival rates; Link & Doherty, 2002).

To investigate the possible relationships between mean survival rates and their EVs we compiled estimated EVs of annual survival rates in five vertebrate taxa (mammals, birds, reptiles, amphibians and fish) from 89 species, ranging from low to high mean survival rates. We only used estimates of EVs that were based on a decomposition of observed variance into sampling and environmental variance in survival rates as sampling variance does not affect the dynamics of populations. We first show how EVs broadly relate to mean annual survival rates among species. Within species, EVs commonly vary between adults and juveniles depending on their respective mean survival rates (see, e.g. Gaillard & Yoccoz, 2003; Sæther & Bakke, 2000). We therefore investigated within‐species linear relationship between juvenile‐ adult EVs and the distribution of adult‐juvenile contrasts in EVs. Second, we investigated the effects of different scalings on the patterns of mean annual survival rate and EVs, using raw, relativized and logit‐scaled variances within and between species. Previous reviews on mean survival rate and their EVs have covered fewer vertebrate species and taxa (e.g. mammals; Gaillard & Yoccoz, 2003; birds; Sæther & Bakke, 2000; Schmutz, 2009), but more importantly have not investigated how different scaling transformations (i.e. relative and especially the logit‐transformed variances) change the general patterns observed within and across species and taxa (but for plants, see McDonald et al., 2017).

## 2 MATERIALS AND METHODS

### 2.1 Data

We extracted all studies up to and including 2018 from Web of Science that cited the following publications describing methods for estimating EV of life history traits, that is, Link and Nichols (1994), Engen et al. (1998), Gould and Nichols (1998), Kendall (1998), White and Burnham (1999), Akcakaya (2002), Burnham and White (2002) and Altwegg et al. (2007). White and Burnham (1999) have a very large number of citations because it is the primary reference for the program MARK. For these studies, we therefore used the additional search terms ‘reproduction AND variance’, ‘survival AND variance’, ‘environmental varia*’, ‘process variance’, ‘sampling variance’, ‘vital rates’ and ‘(reproduction OR fecundity OR fertility) AND variance’ to filter the citations. Since we only found very few studies with data on EVs in reproductive rates we focused only on survival rates. We also included other relevant studies, such as papers referenced in Morris et al. (2011) and data in Morris and Doak (2002).

We only used data from studies that explicitly partitioned observed variance into process and sampling variance. All methods to partition variance considered in this paper are listed in the linked data file, see Data Availability Statement. We furthermore selected studies estimating only mean annual survival rates (studies based on survival during shorter time periods were discarded). Also, we only kept studies where EVs were estimated from models without any environmental covariates (e.g. climate variables or population density) as that would reduce EVs and make them incomparable across species. Using these criteria, we retrieved 69 studies and compiled data on species and population identities, time frame, mean annual values, environmental variance of the traits (accounting for sampling variance), sample size and variance decomposition method used. We restricted our analysis to survival since data on other demographic parameters (mainly fecundity) were relatively few and difficult to compare as studies defined these parameters in different ways.

To investigate the effects of variance constraints on survival rates with means close to zero and one, we considered two commonly used transformations. First, we used relativized variance *V*_rel_ (Gaillard & Yoccoz, 2003; Morris & Doak, 2004) which is the variance of the survival rates divided by the theoretical maximum variance for a random variable restricted to the interval (0, 1):

$$V_{\mathrm{rel}}=\mathrm{var}/\left(\phi \left(1-\phi \right)\right),$$

where *ϕ* denotes the mean survival rate for a stage/age class. Second, we considered the variance of logit‐transformed survival rates. To do this, we used the delta method to approximate the logit‐scaled variance *V*_logit_ (Bjørkvoll et al., 2016; Link & Doherty, 2002) according to

$$V_{\text{logit}}=\mathrm{var}/{\left(\phi \;\left(1-\phi \right)\right)}^{2}.$$

**FIGURE 1:**
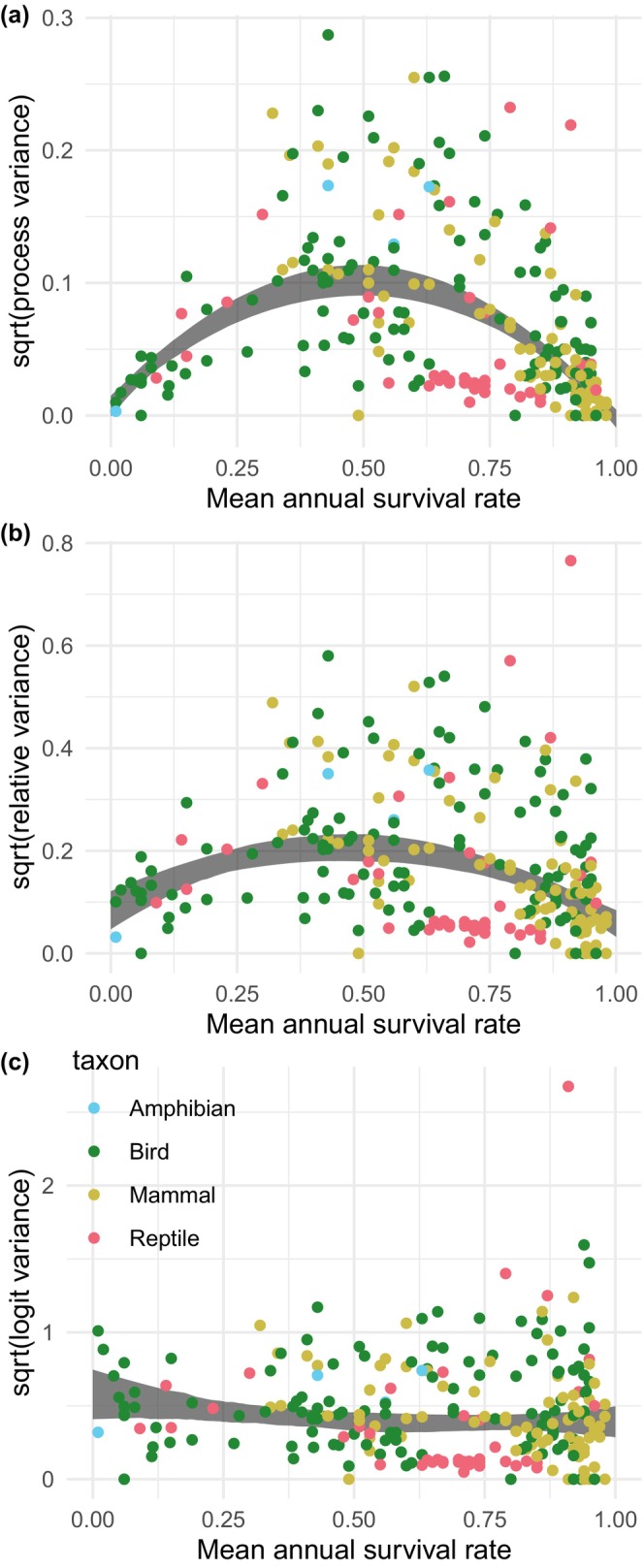
Relationship between environmental variance (expressed as standard deviations) and mean annual survival rate of vertebrates based on (a) arithmetic variances, (b) relativized variances and (c) logit‐transformed variances (see Section 2). The grey area refers to 95% confidence intervals from a quantile regression on median EVs (50% quantile). Colour codes for taxa are shown in (c).

**FIGURE 2:**
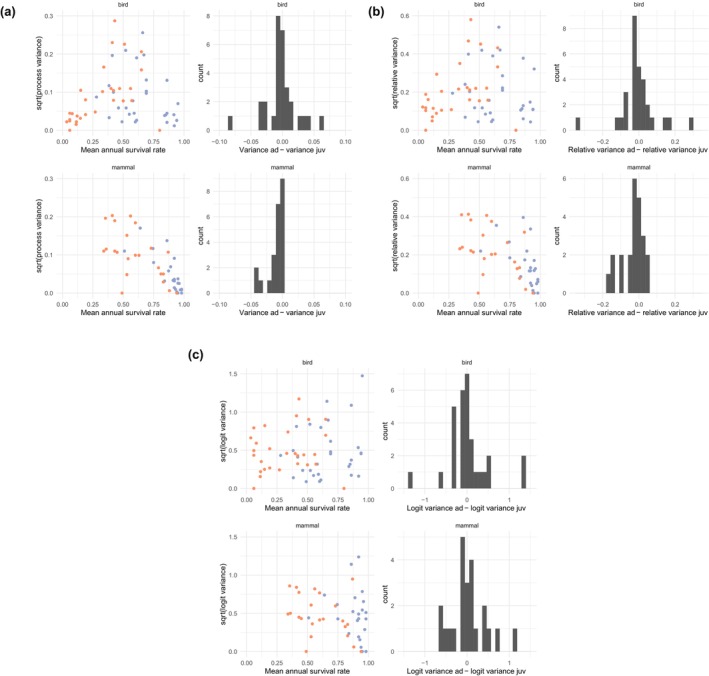
Comparison of juvenile (orange)‐adult (blue) mean survival and EVs (expressed as SD) with lines connecting estimates from the same population, and distributions of adult‐juvenile EV contrast for birds and mammals. (a) Raw EVs, (b) relativized EVs and (c) logit‐transformed EVs.

We visualize the among‐species relationships between mean annual survival and EVs on arithmetic, relativized and logit scales, displayed as standard deviations (sqrt [EV]). We used quantile regression of the square root of EVs against a quadratic function of mean annual survival rates to estimate the relationships (see Figure 1). For the quantile regression, we used the median (50% quantile), and estimated parameters via the quantreg package (Koenker, 2023) in R. We also display within‐species relationships between juvenile and adult mean survival and their EVs by plotting juvenile‐adult slopes and contrasts (see Figure 2). Because mean annual survival rates of immatures or subadults often are close to mean adult survival rates we discarded estimates of EVs for age/stage classes in between juvenile (i.e. first‐year survival) and adults to increase the contrast.

## 3 RESULTS

We found 252 estimates of environmental (process) variance, EV, of survival rates from 89 different species of birds, mammals, reptiles, amphibians and fish (Figure 1). Mean annual survival rates ranged from 0.01 to 0.98 with a bias in our data towards species with high mean adult survival rates (Figure 1a). Mean annual survival rates of birds covered the whole range while mammals were dominated by high mean survival rates (i.e. >0.50; Figure 1a–c). Other taxa (reptiles, amphibians and fish) were only represented by a few data points.

In general, variation in estimated EVs was high especially at mid‐ranges of survival rates resulting in a hump‐shaped distribution of the mean–variance relationship (as estimated by quantile regression), with EVs being small at low and high survival rates and EVs generally larger at intermediate survival rates (Figure 1a). Such a hump‐shaped distribution is expected due to the mathematical restrictions on the variance for fixed means. Similarly, variance‐survival slopes within species conform to such restrictions, with many positive juvenile‐adult slopes (i.e. EVs were higher for adults than juveniles) for EVs of species at the lower end of mean survival rates (i.e. survival rates <0.5, mainly birds) and negative slopes (juvenile EVs were higher than for adults) at the higher end (i.e. survival rates >0.5; birds and mammals; Figure 2a) of the mean survival range.

When transforming the *y*‐axis to relativized EVs, the EV‐survival relationship stayed similar as for the raw data, but with a less marked hump‐shape as shown by a shallower quadratic function (Figure 1b). The within‐species juvenile‐adult slopes were also similar to untransformed data with many positive slopes at low survival rates (i.e. <0.5; birds) and negative slopes at high survival rates (i.e. >0.5; birds and mammals; Figure 2b). However, the patterns of adult‐juvenile slopes were less distinct than for untransformed data.

Although the high variation was maintained in logit‐scaled EVs, these estimates displayed a more stable variation across the whole span of survival rates without showing any clear mean survival rate‐EV relationship (Figure 1c). Similarly, within‐population juvenile‐adult slopes displayed no clear pattern, with negative or positive slopes across the whole range of mean survival rates (Figure 2c).

There was no clear difference in the survival rate‐EV relationships between mammals, birds, reptiles and amphibians covering the same survival rate range, irrespective of the scaling of EVs (Figures 1a–c and 2a–c).

## 4 DISCUSSION

Our compilation of survival rates and their EVs shows a large variation in EVs both within‐species and between‐species characterized by similar mean annual survival rates. However, we also detected a general hump‐shaped relationship between EVs and mean survival rates for untransformed EVs. The within‐population slopes of juvenile‐adult EVs were positive for species with mean survival rates below 0.5 while the corresponding slopes were negative for species with mean survival rates higher than 0.5. Similarly, positive relationships between juvenile and adult EVs in survival have been shown for small mammals characterized by low mean survival rates while these relationships generally were negative for mammals with high mean survival rates (Gaillard & Yoccoz, 2003).

Relativized EVs (which can theoretically take on any value between 0 and 1 regardless of mean survival), retained the hump‐shaped pattern and the patterns of within‐species juvenile‐adult slopes of EVs, although less pronouncedly so. Other studies investigating life history variation and the relationship to EVs of survival rates have investigated the relationships between sensitivities/elacticities of survival and their EV's (e.g. Gaillard & Yoccoz, 2003; Hilde et al., 2020; Schmutz, 2009). These studies also suggest distinct relationships observed on raw data (e.g. negative survival sensitivity–EV relationships from intermediate to high survival rates;) but less distinct relationships when using relativized EVs (Forcada et al., 2008; Gaillard & Yoccoz, 2003; birds: Sæther & Bakke, 2000; Schmutz, 2009).

The logit‐scaled transformation, however, completely changed the survival‐EV relationship to largely display no relationship between annual mean survival rate and EVs and the variation in EVs being more even across the gradient from low to high survival rates (see also juvenile‐adult slopes and contrasts). The few previous studies comparing patterns based on raw or relativized variances to those on a logit‐transformed scale similarly showed marked different or disappearing mean survival‐EV relationships (Bjørkvoll et al., 2016; McDonald et al., 2017). However, which scaling of vital rates is most appropriate for studying EVs in terms of life history evolution is not clear (Link & Doherty, 2002). For example, while relativized EVs remove the dependence of the theoretical maximum variance on the mean, one may question to what extent this scaling is ecologically relevant as it is only attained for extreme cases of variation (survival rates taking values of either 0 or 1; Bjørkvoll et al., 2016). Similarly, how to biologically interpret patterns based on logit‐scaled EVs is also not clear. On the other hand, the more stable variance pattern at the logit scale could suggest that the logit transformation is a suitable candidate for empirical comparisons of survival rates, which, for example, is important when deciding what transformation to use for sensitivity analyses (Link & Doherty, 2002).

There are many possible sources to the observed variation in EVs between and within species characterized by similar mean survival rates (see, e.g. Hilde et al., 2020) and we did not intend to investigate them. However, there is almost always statistical uncertainty in annual survival rate estimates due to incomplete sampling and measurement error (e.g. due to the size and configuration of study areas, see Doligez & Pärt, 2008). The length of time series and temporal autocorrelation in environmental conditions also affect the accuracy of estimates of the mean and variance of survival rates (Hilde et al., 2020). Environmental conditions driving the patterns of EVs are also likely to vary greatly among studies depending on geographic location and local environmental conditions (Hilde et al., 2020). Estimates of EVs may furthermore vary due to the complexity and assumptions of the models used. For example, models using environmental covariates will reduce any estimates of EV and we therefore omitted such studies. However, even models without environmental covariates may produce different estimates of EVs, depending on the model structure and method used to estimate sampling and process variance, but to test such effects on estimates in EVs would require large‐scale simulation of all different model structures and methods used. Finally, if a part of the observed variation in EVs is driven by life history evolution as suggested by Gaillard and Yoccoz (2003) and McDonald et al. (2017); it is also possible that species with similar life histories differ in their response to a specific environment. For example, depending on the type and degree of environmental variation, buffering may be selectively advantageous for one demographic parameter (e.g. adult survival) while lability may be selected for another demographic parameter, for example, juvenile survival (cf. Hilde et al., 2020; Le Coeur et al., 2022). Furthermore, environmental covariances among demographic parameters may also affect EV of a specific demographic parameter (Hilde et al., 2020; Le Coeur et al., 2022). At present, we cannot decompose all these different possible causes of the observed variation in EVs within and between species sharing similar survival rates.

The observed high variation in EVs for similar mean survival rates (Figure 1a–c), and the fact that patterns change depending on the type of scaling of variances suggest two things. First, population viability analyses estimating extinction risks, or modelling of long‐term population trajectories in general, need to consider the uncertainties and variability in estimated EVs and their means. For example, a possible way of doing so could be to conduct a sensitivity analysis by varying the mean and EV within the range observed in Figure 1a and observe how extinction risks or other properties are affected. Second, any tests of demographic buffering or lability hypotheses are likely to be linked to large variability in the estimated relationships between sensitivities/elasticities of vital rates and their EVs. Thus, our compilation suggests there is a need for great care when generalizing patterns of environmental variances in vital rates and we suggest that any testing of demographic buffering or lability hypotheses embrace these large uncertainties in the estimates of the covariation of sensitivities/elasticities of vital rates and their respective EVs.

## AUTHOR CONTRIBUTIONS

**Tomas Pärt:** Conceptualization (lead); data curation (equal); funding acquisition (equal); investigation (equal); methodology (equal); project administration (equal); writing – original draft (lead); writing – review and editing (lead). **Tobias Jeppsson:** Conceptualization (lead); data curation (equal); formal analysis (equal); investigation (equal); methodology (equal); project administration (equal); writing – original draft (lead); writing – review and editing (supporting). **Matthieu Paquet:** Conceptualization (supporting); data curation (equal); formal analysis (equal); methodology (equal); project administration (equal); visualization (lead); writing – original draft (supporting); writing – review and editing (equal). **Debora Arlt:** Data curation (supporting); writing – original draft (supporting); writing – review and editing (supporting). **Ane T. Laugen:** Data curation (supporting); writing – original draft (supporting); writing – review and editing (supporting). **Matthew Low:** Data curation (supporting); writing – original draft (supporting); writing – review and editing (supporting). **Jonas Knape:** Conceptualization (supporting); formal analysis (equal); methodology (equal); writing – original draft (equal); writing – review and editing (equal). **Anna Qvarnström:** Conceptualization (supporting); writing – original draft (equal); writing – review and editing (equal). **Pär Forslund:** Conceptualization (lead); data curation (lead); formal analysis (lead); funding acquisition (equal); investigation (lead); methodology (equal); writing – original draft (lead); writing – review and editing (supporting).

## CONFLICT OF INTEREST STATEMENT

There is no conflict of interest among the authors of this paper.

## Data Availability

All compiled data on mean survival rates and their environmental (process) variance are available after publication on DRYAD https://doi.org/10.5061/dryad.v41ns1s3f.
